# Reproducibility of cerebral blood flow, oxygen metabolism, and lactate and N-acetyl-aspartate concentrations measured using magnetic resonance imaging and spectroscopy

**DOI:** 10.3389/fphys.2023.1213352

**Published:** 2023-09-05

**Authors:** Signe Sloth Madsen, Ulrich Lindberg, Sohail Asghar, Karsten Skovgaard Olsen, Kirsten Møller, Henrik Bo Wiberg Larsson, Mark Bitsch Vestergaard

**Affiliations:** ^1^ Department of Anaesthesiology, Pain and Respiratory Support, Neuroscience Centre, Copenhagen University Hospital–Rigshospitalet, Glostrup, Denmark; ^2^ Functional Imaging Unit, Department of Clinical Physiology and Nuclear Medicine, Copenhagen University Hospital—Rigshospitalet, Copenhagen, Denmark; ^3^ Anesthesiology and Intensive Care, Department of Clinical Sciences, Faculty of Medicine, Lund University, Lund, Sweden; ^4^ Department of Neuroanaesthesiology, Neuroscience Centre, Copenhagen University Hospital–Rigshospitalet, Copenhagen, Denmark; ^5^ Department of Clinical Medicine, Faculty of Health and Medical Sciences, University of Copenhagen, Copenhagen, Denmark

**Keywords:** cerebral blood flow, cerebral metabolic rate of oxygen, cerebral lactate, phase contrast mapping, arterial spin labelling, reproducibility, N-acetyl-aspartate

## Abstract

In humans, resting cerebral perfusion, oxygen consumption and energy metabolism demonstrate large intersubject variation regardless of methodology. Whether a similar large variation is also present longitudinally in individual subjects is much less studied, but knowing the time variance in reproducibility is important when designing and interpreting longitudinal follow-up studies examining brain physiology. Therefore, we examined the reproducibility of cerebral blood flow (CBF), global cerebral metabolic rate of oxygen (CMRO_2_), global arteriovenous oxygen saturation difference (A-V.O_2_), and cerebral lactate and N-acetyl-aspartate (NAA) concentrations measured using magnetic resonance imaging (MRI) and spectroscopy (MRS) techniques through repeated measurements at 6 h, 24 h, 7 days and several weeks after initial baseline measurements in young healthy adults (*N* = 26, 13 females, age range 18–35 years). Using this setup, we calculated the correlation, limit of agreement (LoA) and within-subject coefficient of variation (CoV_WS_) between baseline values and the subsequent repeated measurements to examine the longitudinal variation in individual cerebral physiology. CBF and CMRO_2_ correlated significantly between baseline and all subsequent measurements. The strength of the correlations (R^2^) and reproducibility metrics (LoA and CoV_WS_) demonstrated the best reproducibility for the within-day measurements and generally declined with longer time between measurements. Cerebral lactate and NAA concentrations also correlated significantly for all measurements, except between baseline and the 7-day measurement for lactate. Similar to CBF and CMRO_2_, lactate and NAA demonstrated the best reproducibility for within-day repeated measurements. The gradual decline in reproducibility over time should be considered when designing and interpreting studies on brain physiology, for example, in the evaluation of treatment efficacy.

## 1 Introduction

Measurements of cerebral blood flow (CBF), oxygen consumption and energy metabolism are of considerable interest when studying brain physiology and pathophysiology. Numerous research studies have examined the effects of disease or certain interventions on cerebral physiology (Powers et al., 1985; Liddle et al., 1992; Oshima et al., 2003; Peng et al., 2014; Snyder et al., 2015; Wolters et al., 2017; Younis et al., 2021; Vestergaard et al., 2022). However, cerebral physiology, both CBF and energy metabolism, demonstrates very large intersubject variability, which makes it difficult to observe small effects of intervention or disease. Multiple factors modulate CBF both acutely and over longer time periods (Kety and Schmidt, 1948; Lassen, 1959; Iida et al., 2004; Coles et al., 2006; Henriksen et al., 2013); for example, changes in arterial carbon dioxide or oxygen tension as well as oxygen saturation affect CBF (Paulson et al., 1990; Vestergaard et al., 2016; Vestergaard and Larsson, 2019). The cerebral metabolic rate of oxygen (CMRO_2_) has similarly a large intersubject variability but demonstrates smaller acute fluctuations than CBF (Iida et al., 2004; Coles et al., 2006; Peng et al., 2014; Vestergaard et al., 2020) A key role of the regulation of CBF is to maintain CMRO_2_ at constant levels.

Studies have examined intersubject variation in cerebral physiology, but normal longitudinal intrasubject variation is much less examined and has mainly focused only on CBF. However, longitudinal studies on cerebral physiology, for example, in terms of disease progression or intervention, require knowledge of normal longitudinal intrasubject variation for correct scaling of the project and interpretation of the results. Studies have examined the reproducibility of CBF using various techniques, but typically only two measurements were acquired, often with only a short time between scans (hours or a few days).

In the present study, we aimed to measure intrasubject variation in cerebral physiology by performing multiple repeated measurements in the same subjects with 6 h, 1 day, 7 days and several weeks between examinations. We used MRI techniques to acquire brain physiological parameters. Due to the noninvasiveness of MRI, it is possible to obtain multiple repeated measurements with only minor considerations related to patient safety. For example, exposure to radiation is not a concern, as would be the case if using positron emission tomography (PET) imaging. MRI techniques were therefore chosen as the most suitable examination method. Global average CBF and CMRO_2_ were acquired using phase contrast mapping (PCM) and susceptibility-based oximetry (SBO) MRI. CBF maps were obtained using the arterial spin labelling (ASL) MRI technique.

In addition to CBF and CMRO_2_, we also measured the concentrations of cerebral lactate and N-acetyl-aspartate (NAA) by magnetic resonance spectroscopy (MRS). In the healthy brain, most of the glucose for energy production is fully oxidized, resulting in an oxygen-to-glucose ratio close to 6. However, in the young healthy brain, approximately 10% of glucose goes through oxygen-free glycolysis with lactate as the end-product (Goyal et al., 2014; Hyder et al., 2016). By measuring the cerebral lactate concentration, we can examine the consistency of this glycolytic activity over time. NAA is predominantly synthesized in neurons and is primarily a marker of neuronal density. The NAA concentration is lower in patients with dementia and is reduced after stroke where it correlates with neuron loss. However, NAA is also affected by neuronal metabolic activity. For example, during disease activity in multiple sclerosis and after traumatic brain injury, the NAA concentration is reduced. Yet, this reduction is reversed during recovery. This suggests that NAA concentration is also affected by metabolic integrity, in addition to neuronal density. (Bitsch et al., 1999; Moffett et al., 2007). In healthy subjects, as examined in the current study, the NAA concentration is expected to be stable, however knowing the normal variation is important to correctly use NAA as a marker of neuronal function.

Overall, we examined intrasubject longitudinal variation in CBF, CMRO_2_, arteriovenous oxygen saturation difference (A-V.O_2_), cerebral lactate concentration, and cerebral NAA concentration in healthy humans through repeated MRI scan sessions. By measuring all these parameters, we obtained a comprehensive examination of longitudinal intrasubject variation in several markers of cerebral physiology.

## 2 Materials and methods

### 2.1 Subjects

Two groups of young healthy subjects participated in the study. All participants were healthy, right-handed, non-smokers, with a body mass index of 18–30 kg/m^2^ and normal physical and neurological examinations. Female participants were non-pregnant. The participants were instructed to have no intake or use of caffeine, alcohol, medication or other substances known to influence the parameters measured in the study prior to study participation. Detailed description regarding the inclusion criteria of the participants are provided in Madsen et al. (2020). In the first group (Group A), ten young healthy subjects (5 females) with a mean age of 25.4 years (range 18–35 years) were included and examined in four MRI sessions with repeated measurements acquired at 6 h, 1 day, and 7 days after an initial baseline measurement. All participants adhered to the same schedule, aligning the weekdays and timing of all scans and measurements. All participants were fasting 2 h for clear liquids and 6 h for all other intake before baseline scans. Between baseline and the second scan on the same day, the participants were served a light meal and drinks adhering to protocol (e.g., no caffeine, alcohol or medication). Next, we examined a second group (Group B), which included sixteen young healthy subjects (8 females) with a mean age of 23.9 years (range 18–32 years) who were examined twice with 28–49 days (mean 31.9 days) between examinations. Thus, we investigated short-term repeatability (up to 7 days) in Group A and repeatability after several weeks in Group B. Group B was included as a follow-up analysis due to the highly stable correlations observed in Group A, and we wanted to examine whether correlations were maintained after several weeks. All participants in Group B also adhered to a strict schedule, aligning the weekdays and timing of all scans and measurements, and following the same fasting regime for all scans used in this study. The participants from Group B were part of a study on the effect of general anaesthesia on brain structure and physiology; none of these subjects underwent anaesthesia during the present study (Madsen et al., 2020). Data from Group A was acquired in 2019, and data from Group B was acquired subsequently, in 2019–2021.

**FIGURE 1:**
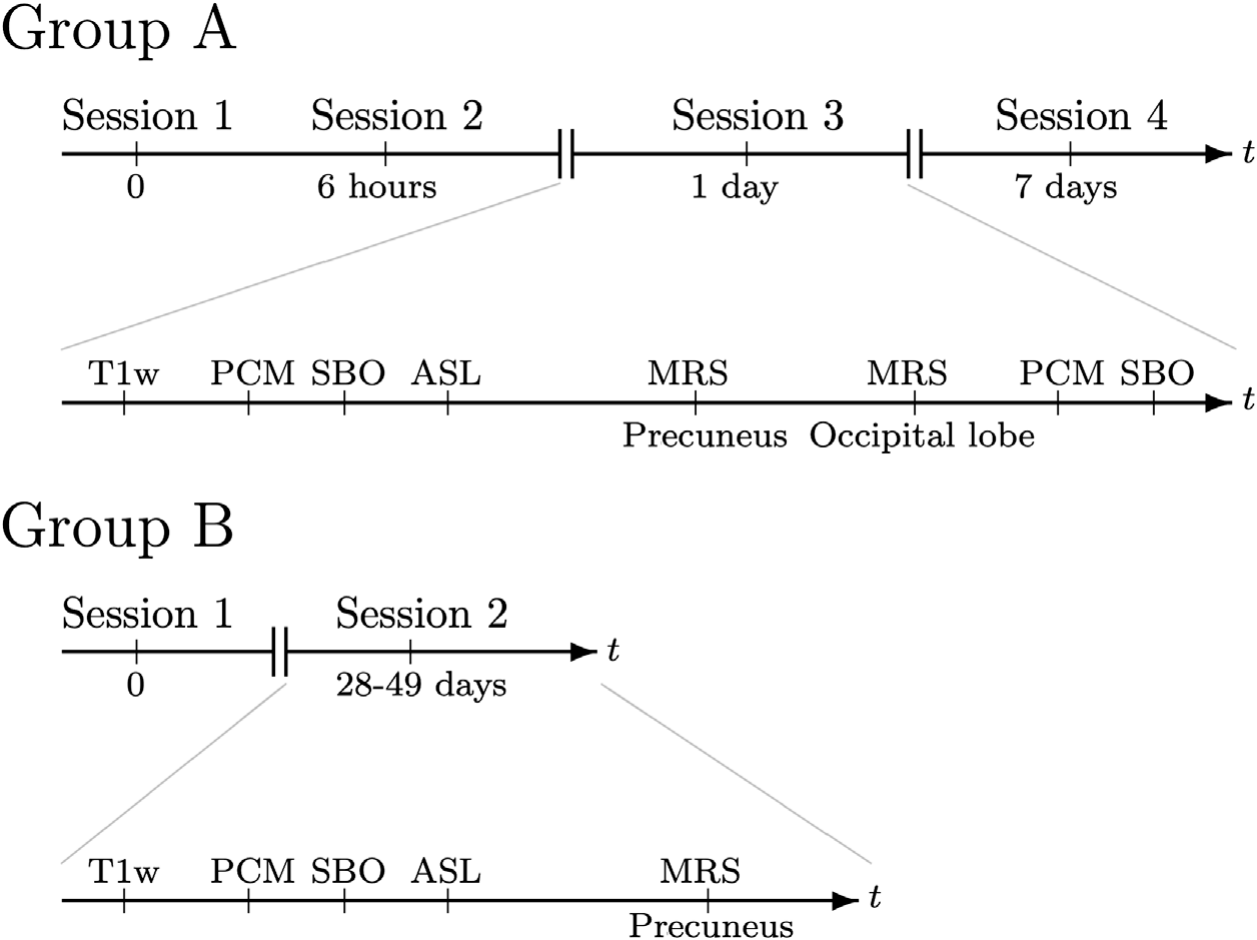
Outline of the MRI sessions and acquisition of cerebral physiology parameters allowing for examination of reproducibility. Group A underwent MRI scans four times, with 6 h, 1 day and 7 days between reacquisitions after an initial baseline measurement. Group B underwent MRI scans two times, with the second scan 28–49 days after the initial baseline scan. In each session, structural brain images were acquired using a T1-weighted high-resolution anatomical MRI sequence (T1w). Cerebral blood flow (CBF) was measured using phase contrast mapping (PCM) and arterial spin labelling (ASL) MRI techniques. Susceptibility-based oximetry (SBO) was used to measure the venous oxygen saturation of the blood leaving the brain, from which the cerebral metabolic rate of oxygen (CMRO_2_) was calculated. Cerebral NAA and lactate concentrations were measured using magnetic resonance spectroscopy (MRS).

In all sessions, we measured global CBF and CMRO_2_ twice (run 1 and run 2) at the beginning and end of the session to determine within-session reproducibility of these measurements. The remaining parameters were measured once in each session. The study setup is depicted in Figure 1.

Participants were recruited through advertisement on a Danish recruitment website (www.forsøgsperson.dk). Exclusion criteria were known neurological diseases and contraindications for MRI, such as metal implants and recent operations or pregnancy. The study was approved by the scientific ethical committee of the Capital Region of Denmark (H-18020364 and H-18028925) and was carried out according to the Declaration of Helsinki.

### 2.2 Magnetic resonance imaging

All scans were acquired on a 3 T Philips (Philips Medical Systems, Best, Netherlands) Achieva dStream (software release 5.4.1) equipped with a 32-channel phased-array receive head coil. The scanner was subjected to ongoing maintenance from the manufacture (Philips Healthcare); and we performed quality control and monitored the scanner and coil performance every week. There were no major software updates or hardware changes during data collection for this study.

The heart rate, arterial oxygen saturation and end tidal CO_2_ partial pressure (PetCO_2_) were measured continuously throughout the MRI scans using a Veris Monitor system (MEDRAD, Pittsburgh, Pennsylvania, USA). The blood pressure was measured with regular intervals during the examinations. The average values of these measurements throughout the scans are presented in Table 1. A venous blood sample was drawn before each scan session and analysed for haemoglobin (Hgb) concentration using a blood gas analyser XN 9000 (Sysmex, Kobe, Japan).

**TABLE 1:** Summary of the acquired parameters from each MRI session. The means ± standard deviations are noted. Abbreviations: A-V.O_2_, arteriovenous oxygen saturation difference; CBF, cerebral blood flow; CMRO_2_, cerebral metabolic rate of oxygen; NAA, N-acetyl-aspartate.

	Group A				Group B	
	Baseline	6 hours	1 day	7 days	Baseline	28–49 days
**Phase Contrast Mapping**						
CBF (Whole brain mean) [ml/100 g/min]	57.5 ± 8.3	58.4 ± 8.6	55.3 ± 6.3	56.9 ± 7.0	62.5 ± 7.8	62.8 ± 7.2
**Arterial Spin Labelling**						
CBF (Grey matter mean) [ml/100 g/min]	59.2 ± 13.4	57.7 ± 13.1	56.3 ± 9.1	63.3 ± 11.8	67.7 ± 10.7	68.1 ± 8.9
**Susceptibility-Based Oximetry**						
A-V.O_2_ [%]	28.7 ± 5.2	29.3 ± 5.5	27.1 ± 6.1	29.6 ± 5.8	29.4 ± 6.3	29.3 ± 3.8
CMRO_2_ [µmol/100 g/min]	143.2 ± 32.6	145.9 ± 30.6	128.7 ± 32.0	148.4 ± 38.6	156.4 ± 36.2	156.4 ± 24.7
**MR Spectroscopy**						
Lactate—precuneus [mmol/l]	0.51 ± 0.12	0.49 ± 0.11	0.50 ± 0.14	0.49 ± 0.10	0.50 ± 8.5	0.52 ± 12.3
Lactate—occipital lobe [mmol/l]	0.51 ± 0.12	0.49 ± 0.11	0.48 ± 0.11	0.52 ± 0.10		
NAA—precuneus [mmol/l]	10.1 ± 2.0	9.7 ± 1.5	9.7 ± 1.5	10.1 ± 1.5	9.4 ± 2.1	9.3 ± 2.1
NAA—occipital lobe [mmol/l]	8.2 ± 2.4	8.2 ± 2.6	7.7 ± 2.1	8.2 ± 2.5		
**Cardiovascular parameters**						
Haemoglobin concentration [mmol/l]	9.1 ± 1.2	9.0 ± 1.1	9.0 ± 1.2	9.1 ± 1.2	8.7 ± 0.9	8.6 ± 0.9
Arterial saturation [%]	97.5 ± 1.6	97.0 ± +0.7	97.7 ± 1.0	97.2 ± 1.4	97.9 ± 1.2	97.9 ± 0.7
Mean arterial blood pressure [mmHg]	88.3 ± 3.6	90.3 ± 3.7	88.6 ± 7.5	86.7 ± 5.7	92.1 ± 8.4	86.2 ± 6.6
End-tidal CO_2_ partial pressure [kPa]	4.7 ± 1.2	5.1 ± 0.5	4.9 ± 0.5	4.9 ± 0.5	4.8 ± 0.7	4.7 ± 0.8
Heart rate [bpm]	59.8 ± 5.4	62.8 ± 5.8	61.4 ± 5.9	63.8 ± 5.6	64.0 ± 9.7	59.8 ± 10.0

#### 2.2.1 Structural images

Structural brain images were acquired using a sagittal three-dimensional T1-weighted high-resolution Magnetization Prepared Rapid Gradient Echo (MPRAGE) scan (echo time (TE) = 2.8 ms, repetition time (TR) = 6.9 ms, inversion time (TI) = 900 ms; flip angle = 9°, 137 slices, field-of-view (FOV) = 262 × 280 × 150 mm^3^; voxel size = 1.1 × 1.1 × 1.1 mm^3^). Bias field correction and segmentation of the brain into grey matter, white matter and cerebrospinal fluid (CSF) was carried out using FAST (FSL 5.0.11, FMRIB, Oxford, UK) (Jenkinson et al., 2012) to estimate brain volume. The anatomical images were additionally used for structural normalisation for the ASL analysis.

#### 2.2.2 Arterial spin labelling (ASL)

Cerebral blood flow maps were acquired using arterial spin labelling (ASL). A dual-echo two-dimensional echo planar imaging pseudo-continuous arterial spin labelling (pCASL) sequence scan with 2 background suppression pulses was used (16 transverse slices (6 mm thick); TE_1_/TE_2_ = 12.56/31.66 ms; TR = 4550 ms (for one image); flip angle = 90°; FOV = 240 × 240 mm^2^; matrix size = 88 × 88 (acquired), 128 × 128 (reconstructed); labelling duration = 1800 ms; postlabelling delay = 1800 ms with an additional slice acquisition time of 32 ms; background suppression (BS) pulses = BS_1_/BS_2_: 1813/3135 ms; 60 dynamics, 30 label/control pairs). A calibration scan was acquired with the same imaging parameters using a 16-phase look-locker readout after saturation (saturation delay (TD) = 50 ms; TR = 520 ms; slice timing difference = 32 ms; flip angle = 30°; 2 dynamics). R1 (relaxation rate R1 = 1/T1) was fitted from the saturation recovery signal using Eq. 1 by in-house developed MATLAB (version 9.10.0 (R2021a), The MathWorks Inc, Natick, Massachusetts, United States) programs utilizing a nonlinear least squares Levenberg‒Marquardt method.

$$S\left(t\right)=S_{0}\sin\left(\alpha \right)\left[\left(1-e^{-TD∙R_{1}}\right){\left(\cos\left(\alpha \right)e^{-TR∙R_{1}}\right)}^{n-1}+\left(1-e^{-TR∙R_{1}}\right)\frac{1-{\left(\cos\left(\alpha \right)e^{-TR∙R_{1}}\right)}^{n-1}}{1-\cos\left(\alpha \right)e^{-TR∙R_{1}}}\right]$$ (1)

TD is the time since saturation, corrected for individual slice timing; TR is the time interval between two consecutive alpha pulses; and n is the index of the alpha pulses, which for this sequence took the values *n* = [1:16]. The R_1_ value was used to calculate M_0_ from the initial six pCASL control measurements using Eq. 2.

$$M_{0}=\frac{\frac{1}{6}\sum_{j=1}^{6}M_{ctr,j}}{\left[\left(1-e^{-R_{1}\left(TD-{BS}_{2}\right)}\right)+\left(-\left(1-e^{-R_{1}\left({BS}_{2}-{BS}_{1}\right)}\right)+\left(1-e^{-R_{1}{BS}_{1}}\right)e^{-R_{1}\left({BS}_{2}-{BS}_{1}\right)}\right)e^{{-R}_{1}\left(TD-{BS}_{2}\right)}\right]}$$ (2)

BS_1_/BS_2_ are the timing of the background suppression pulses and M_ctr_ is the control part of the ASL data pairs. TD is the read-out time after saturation, corrected for slice timing differences.

TD=3.6s+slicedt∙i,where i=0,1,⋯15

The perfusion weighted maps were converted to quantitative CBF maps using the voxel-wise M_0_ signal from Eq. 2 in the BASIL tool (FSL) (Chappell et al., 2009) with additional correction of tissue T_1_-and R_2_*-decay. T_1_ of blood was corrected for each subject’s session-specific haemoglobin concentration using the arterial equation from Lu et al. (Lu et al., 2004). Grey matter mean CBF values were extracted from the grey matter segmentations of the structural images resliced into ASL space using the oxford_asl (part of FSL) tool with the default threshold parameter of 0.8.

#### 2.2.3 Phase contrast mapping

Global mean CBF was acquired using the PCM technique by measuring the blood flow supplying the brain through the carotid and basilary arteries (Bakker et al., 1995; Vestergaard et al., 2017). Velocity maps were acquired by a velocity-encoding turbo field echo sequence (1 slice, FOV = 240 × 240 mm^2^; voxel size = 0.75 × 0.75 × 8 mm^3^; TE/TR = 7.33/27.63 ms; flip angle = 10°; velocity encoding = 100 cm/s, without cardiac gating; 10 dynamics). The sequence was recorded twice to obtain optimal perpendicular slice positions on both internal carotid arteries (first scan) and the basilar artery (second scan). Each feeding artery was manually delineated using in-house developed MATLAB scripts. The blood flow to the brain was then calculated as the mean blood velocity times the cross-sectional area of the delineated cerebral arteries. The resulting flow was normalised to brain weight to obtain values in ml/100 g/min. Brain weight was estimated from the segmentation of the structural MRI image, including grey matter and white matter but excluding CSF and assuming a brain density of 1.05 g/ml (Torack et al., 1976).

#### 2.2.4 Susceptibility-based oximetry (SBO)

Cerebral arteriovenous oxygen saturation differences (A-V.O_2_) and CMRO_2_ were acquired using susceptibility-based oximetry (SBO) MRI (Jain et al., 2010). Using the SBO technique, the oxygen saturation of the venous blood (SvO_2_) leaving the brain in the sagittal sinus can be measured. The technique utilizes the magnetic properties of deoxyhaemoglobin in venous blood changes the intravascular magnetic susceptibility, which can be measured by MRI phase images. Susceptibility-weighted phase maps were acquired using a dual-echo gradient-echo sequence (1 slice, FOV = 220 × 190 mm^2^; voxel size = 0.69 × 0.69 × 8 mm^3^; TR = 23.1 ms; TE_1_/TE_2_ = 8.16/17.83 ms; flip angle = 30°; SENSE factor = 2; 10 dynamics, velocity encoding = 100 cm/s). The imaging plane was placed orthogonal to the sagittal sinus. By manual delineation of the sagittal sinus and the surrounding tissue, SvO_2_ was calculated. An in-depth description of the postprocessing has been previously published (Vestergaard and Larsson, 2019). A-V.O_2_ was calculated by subtracting SvO_2_ from arterial saturation (SaO_2_) measured using pulse oximetry. CMRO_2_ was then calculated using the Fick principle (Eq. 3). Haemoglobin (Hgb) concentrations were measured from venous blood sampling.

$${CMRO}_{2}=Hgb\cdot CBF\cdot A-V.O_{2}$$ (3)

From this sequence, we simultaneously also acquired phase information from which we could calculate the blood flow in the sagittal sinus by a similar approach as that used for calculating the flow in the feeding cerebral arteries by manual delineation of the sagittal sinus.

#### 2.2.5 Magnetic resonance spectroscopy (MRS)

Cerebral NAA and lactate concentrations were measured using a single-voxel water-suppressed point-resolved 1H-spectroscopy (PRESS) sequence (TE/TR = 288/2000 ms; voxel size = 30 × 35 × 30 mm^3^; 176 averages, 1,024 complex data points). Precuneus is a part of the default mode network and relatively metabolic stable, whereas the occipital cortex is a region which possibly could demonstrate more metabolic variation. These two regions could therefore possibly demonstrate different reproducibilities. For Group A, two measurements were acquired, one in the precuneus and a second in the occipital lobe. For Group B, only measurements in the precuneus were acquired due to the similar concentrations in the precuneus and occipital lobe observed in Group A.

Postprocessing and quantification of the spectra were performed using LCModel (LCModel, Version 6.3-1F, Toronto, Canada). The water peak acquired in the spectrum was used as reference to the measured metabolites. The water concentration in the spectroscopy voxel was estimated from the content of grey matter, white matter, and CSF within the voxel using the tissue segmentations from the structural images (Quadrelli et al., 2016). NAA and lactate concentrations were corrected for T_2_ decay using literature values (T_2,H2O_ = 95 ms; T_2,NAA_ = 247 ms; T_2,Lac_ = 240 ms) (Wansapura et al., 1999; Träber et al., 2004).

### 2.3 Statistics

All values are reported as means ± standard deviations. *p*-values less than 0.05 were considered significant. Reproducibility between the baseline measurement and subsequent repeated measurements was assessed by linear regression models and Bland‒Altman analysis. R^2^ values from the regression models and limit of agreement (LoA) metrics and within-subject coefficient of variation (CoV_ws_) from Bland‒Altman analysis were used to assess the reproducibility of the measurements. LoA was calculated as 1.96 times the mean standard deviation of the pairwise differences between baseline and each subsequent measurement.

### 2.4 Data availability

The parameters derived from the MRI-images and supporting data are available upon reasonable request. The MRI images are not publicly available due to privacy restrictions.

### 2.5 Code availability

Software used for calculating CBF by the PCM technique is available at https://github.com/MarkVestergaard/PCMCalculator/. Software used to calculate SvO_2_ from the SBO technique is available at https://github.com/MarkVestergaard/SBOCalculator/.

## 3 Results

For one subject in Group A, ASL analysis at the baseline measurement failed and this subject was removed from the further analysis of ASL reproducibility. Furthermore, the ASL analysis failed in one subject at the 6-h measurement and two subjects at the 7-day measurement in Group A. The failure to analyse these ASL measurements was due to technical issues with the calibration scan. For the other parameters, all data were satisfactorily acquired.

The average values of the acquired metrics in each MRI session are provided in Table 1. A summary of R^2^ values from the linear regression, LoA from Bland‒Altman analysis and CoV_ws_ for all parameters and correlations are summarized in Table 2. There were no significant differences in baseline values between group A and B.

**TABLE 2:** Summary of R^2^, limit of agreement (LoA) and within-subject coefficient of variation (CoV_ws_) for the correlations between baseline and the subsequent measurements. Limit of agreement is defined as 1.96 standard deviations from Bland–Altman analysis. Abbreviations: A-V.O_2_, arteriovenous oxygen saturation difference; CBF, cerebral blood flow; CMRO_2_, cerebral metabolic rate of oxygen; NAA, N-acetyl-aspartate.

	Group A												Group B		
	1 hour (within-scan session)			6 hours			1 day			7 days			28–49 days		
	*N* = 40			*N* = 10			*N* = 10			*N* = 10			*N* = 16		
	R^2^	LoA	CoV_ws_ [%]	R^2^	LoA	CoV_ws_ [%]	R^2^	LoA	CoV_ws_ [%]	R^2^	LoA	CoV_ws_ [%]	R^2^	LoA	CoV_ws_ [%]
**Phase Contrast Mapping**															
CBF (Whole brain mean) [ml/100 g/min]	0.84	8.1	7.4	0.86	6.3	5.6	0.71	8.9	8.1	0.57	10.6	9.5	0.31	13.8	11.2
**Arterial Spin Labelling**															
CBF (Grey matter mean) [ml/100 g/min]				0.85	10.7	9.3	0.71	14.6	12.8	0.69	16.7	13.9	0.31	18.3	13.1
**Susceptibility-Based Oximetry**															
A-V.O_2_ [%]	0.87	4.0	7.0	0.57	7.7	13.7	0.47	9.12	16.7	0.26	10.7	18.6	0.29	12.4	19.1
CMRO_2_ [µmol/100 g/min]	0.89	23.8	8.8	0.55	45.6	16.2	0.43	52.74	20.0	0.56	49.9	17.5	0.29	60.9	19.8
**MR Spectroscopy**															
Lactate—precuneus [mmol/l]				0.79	0.11	11.0	0.45	0.21	20.9	0.10	0.25	25.9	0.50	0.17	17.0
Lactate—occipital lobe [mmol/l]				0.51	0.17	17.4	0.41	0.19	19.4	0.32	0.21	20.4			
NAA—precuneus [mmol/l]				0.87	1.78	11.0	0.39	3.87	19.2	0.45	3.89	24.3	0.75	2.08	11.2
NAA—occipital lobe [mmol/l]				0.81	1.83	9.5	0.87	1.57	8.1	0.77	1.94	9.8			

**FIGURE 2:**
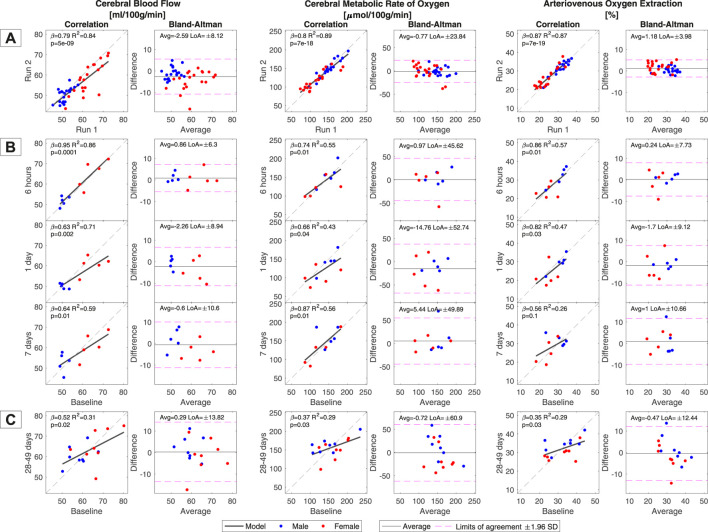
Correlation and reproducibility between baseline measurements and the subsequent acquisitions of global cerebral blood flow (CBF), cerebral metabolic rate of oxygen (CMRO_2_) and arteriovenous oxygen saturation difference (A-V.O_2_). **(A)** The within-session correlation was calculated from the duplicate measurements of CBF, A-V.O_2_ and CMRO_2_ that were acquired in each MRI session. The correlations between baseline and values from each subsequent MRI from Group A are shown in **(B)**. The correlations between baseline and values remeasured 28–49 days after the initial examination in Group B are shown in **(C)**. A general pattern of weaker correlation with increasing time between sessions is noted. Limits of agreement (LoAs) in the Bland–Altman analysis were calculated as 1.96 standard deviations of the pairwise subtracted values. Sex was not part of the regression model but is highlighted for visual interpretation. The regression slopes (β), R^2^ coefficients, *p*-values and LoAs are noted in each panel.

The correlations and Bland‒Altman analysis for global CBF, CMRO_2_ and A-V.O_2_ are demonstrated in Figure 2. The correlations for CBF, CMRO_2_ and A-V.O_2_ were significant for all comparisons. Correlations and reproducibilities were best for within-day measurements for all parameters (LoA = 6.3 ml/100 g/min; CoV_ws_ = 5.6% for CBF and LoA = 45.6 µmol/100 g/min, CoV_ws_ = 16.2% for CMRO_2_) and gradually declined for longer time periods between measurements. The within-session reproducibility of CBF and CMRO_2_ (Figure 2) demonstrated good reproducibility with low LoA (8.1 ml/100 g/min and 23.8 µmol/100 g/min) and highly significant correlations (R^2^ = 0.84 and 0.89). However, CBF also demonstrated a significant bias towards lower values for the second measurement in the session compared to the first measurement (first measurement = 57.5 ± 8.3 ml/100 g/min; second measurement = 54.4 ± 7.2 ml/100 g/min, *p* = 0.0003).

Global CBF values across all sessions were significantly correlated with both haemoglobin concentration (*p* < 10^–4^) and A-V.O_2_ (*p* = 0.006) (Supplementary Figure S1). Reproducibility of the venous blood flow leaving the brain in the sagittal sinus is presented in Supplementary Figure S2A–C. The blood flow in the sagittal sinus demonstrated similar reproducibility to CBF, including a bias towards lower values at the second compared to the first measurement during the baseline session. There was also a significant correlation between CBF and blood flow in the sagittal sinus (Supplementary Figure S2D).

**FIGURE 3:**
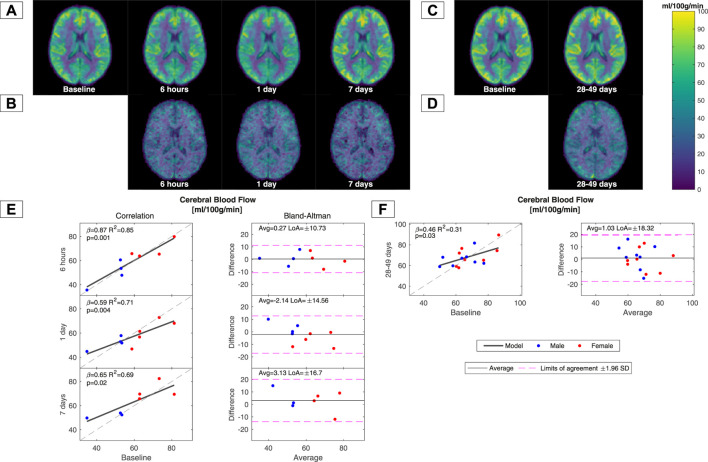
CBF maps acquired using arterial spin labelling and correlations of grey matter mean values between baseline and subsequent measurements. Average CBF maps of all subjects in Group A for each session in MNI-152 standard space are shown in **(A)**. Maps demonstrating voxel-wise limits of agreement (LoAs) between the baseline and subsequent measurements in Group A are shown in **(B)**. Average CBF maps from all subjects in Group B at the two MRI sessions are shown in **(C)**. Maps demonstrating voxel-wise LoAs between the baseline measurement and subsequent measurements 28–49 days after the initial examination of Group B are shown in **(D)**. Correlations and Bland‒Altman analysis between the grey matter mean CBF values from the ASL measurement at baseline and subsequent sessions are shown in **(E)** for Group A and in **(F)** for Group B. The regression slopes (β), R^2^ coefficients, *p*-values, and limits of agreement (LoAs) are noted in each panel.

The mean regional CBF maps from the ASL acquisition and the correlation between the grey matter mean CBF from each session are shown in Figure 3. No significant difference was observed between each session. The voxel-wise LoA maps also did not demonstrate any regional differences in reproducibility for measurements up to 7 days apart. For measurements taken 28–49 days apart, the LoA was generally higher in the cortex than in the rest of the brain. Grey matter mean CBF values from the ASL measurement demonstrated the best reproducibility for within-day measurements and worse reproducibility for the remaining sessions, similar to global CBF values obtained by the PCM method.

Measurements of brain size and changes in haemoglobin concentration will affect the calculation of CBF and CMRO_2,_ and we therefore examined the reproducibility of these measurements as well (Supplementary Figure S3). Both estimation of brain size and haemoglobin concentration demonstrated very high reproducibility and therefore only minorly affected the longitudinal variant in CBF and CMRO_2_ observed in this study. CBF was higher in women across all measurements as also demonstrated in multiple previous studies (Rodriguez et al., 1988; Esposito et al., 1996). There were no sex differences for the remaining parameters.

**FIGURE 4:**
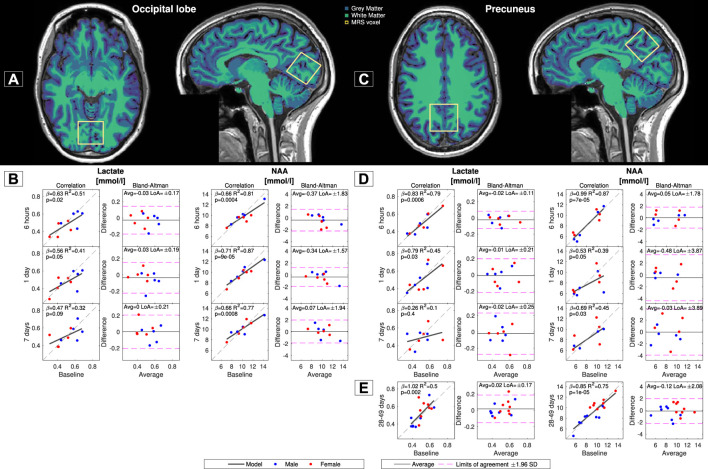
Correlation and reproducibility between baseline measurements and the subsequent acquisitions of cerebral lactate and N-acetyl-aspartate (NAA) concentrations. Lactate and NAA concentrations were measured in the occipital lobe **(A)** and precuneus **(C)**. Correlations and Bland‒Altman analysis of the lactate and NAA concentrations in the occipital lobe between baseline and the subsequent acquisitions from Group A are shown in **(B)** and those from Group B in **(E)**. Correlations and Bland‒Altman analysis of the lactate and NAA concentrations in the precuneus between baseline and the subsequent acquisitions from Group A are shown in **(D)** and those from Group B in **(E)**. The regression slopes (β), R^2^ coefficients, *p*-values from the regressions and limits of agreement (LoAs) from Bland‒Altman analysis are noted in each panel.

The correlations and Bland‒Altman analysis of lactate and NAA concentrations are shown in Figure 4. All comparisons demonstrated significant correlations between baseline and subsequent measurements, except for lactate measurements after 7 days, which were only near significant for the measurement in the occipital lobe (*p* = 0.09) and nonsignificant for the measurement in the precuneus (*p* = 0.40). The reproducibility was best for the within-day measurements for both lactate and NAA and similarly worse for the longer time periods between measurements. The quality parameters for the MRS data were signal-to-noise (SNR) = 38.8 ± 4.4, full width at half maximum (FWHM) = 0.05 ± 0.01, Cramer-Rao lower bounds (CRLB) for lactate = 25.9 ± 10.8%, CRLB for NAA = 2.9 ± 1.1% for the measurements in the occipital lobe and SNR = 42.5 ± 2.7, FWMH = 0.04 ± 0.01, CRLB for lactate = 20.2 ± 3.9% and CRLB for NAA = 2.1 ± 0.4% for the measurements in precuneus.

## 4 Discussion

We observed that repeated measurements of CBF, A-V.O_2_, CMRO_2_, lactate and NAA correlated and demonstrated good reproducibility, including up to several weeks between measurements. For CBF and CMRO_2,_ the reproducibility was best for within-day measurements and gradually declined for longer times between measurements. The reproducibility of lactate and NAA in the occipital lobe was similarly best for within-day measurements and gradually declined for the repeated measurements after 1 and 7 days. For lactate and NAA in the precuneus, the reproducibility was again best for within-day measurements and was equally worse for the remaining repeated measurements.

The variation in repeated measurements is caused by both measurement errors from the equipment and physiological changes. Modifications to the MRI scanner could influence the performance and affect the measurement error. Alterations to the MRI scanner, such as updating the scanner software or replacing the MRI coils, might also modify the reproducibility. However, there were no such modifications on the MRI scanner in the current study, and we do not anticipate the scanner to have significantly deteriorated during the project duration. Overall, we believe that the measurement error of the MRI scanner was similar in all MRI sessions, and we do not expect that any systematic changes occurred. Consequently, we think that the gradual decline in reproducibility we noticed with longer spans between measurements is due to physiological variation. The decreased reproducibility is therefore expected to be similar when using modalities other than MRI scans.

### 4.1 CBF

The techniques we used to measure CBF are noninvasive and widely available on MRI scanners, making them ideal for studies requiring multiple repeated measurements. Both PCM and ASL are therefore extensively used in research on brain physiology. We found CBF values similar to previously reported values using the same methods.

Cerebral perfusion is dynamically regulated to ensure sufficient delivery of nutrients, most notably oxygen, to the brain. Numerous factors affect CBF, for example, changes in blood gas tension, such as oxygen and CO_2_; haemoglobin concentration; and hormonal variations (Kety and Schmidt, 1948; Henriksen et al., 2013; Vestergaard and Larsson, 2019). Intake of certain compounds, such as caffeine or nicotine, will also affect CBF (Zubieta et al., 2005; Krause et al., 2006). Therefore, it is to be expected that CBF will also vary within subjects and knowing the reproducibility over different time periods is important when studying CBF. Generally, we observed better reproducibility for shorter periods between examinations, with the lowest LoA and best correlation for the within-session measurements. However, we also observed a small but significant bias towards lower CBF at the second measurement in the within-session examination, also resulting in a relatively high CoV_ws_. There were approximately 60 min between these two measurements. Interestingly, this lower CBF did not reflect changes in CMRO_2_, suggesting that the effect on CBF from lying in the scanner is not due to changes in cerebral energy metabolism but rather an effect directly on CBF. An effect on CBF from lying for a prolonged time in an MRI scanner should therefore be considered when designing experiments on brain physiology with interventions in the scanner. Prior studies on the reproducibility of CBF have often included just two repeated examinations; therefore, the time variant effects could not be investigated. However, studies on within-day or within-session repeated measurements have generally shown good reproducibility, and studies using longer periods between examinations have similarly shown poorer reproducibility (Fan et al., 2016). A previous study on the reproducibility of CBF measurement by PCM demonstrated slightly worse reproducibility for within-session and within-day examinations compared to our study and similar reproducibility for between-day examinations (Spilt et al., 2002). Another study using 7 weeks between measurements and the ASL technique for the measurement of CBF found reproducibility for whole-brain values similar to our data (Hermes et al., 2007). A study using PET imaging with oxygen-15-labelled water [^15^O-H_2_O] as the radiotracer and a study using ASL MRI found slightly worse within-session reproducibility compared to our study (Coles et al., 2006; Gevers et al., 2009). A study comparing CBF values obtained from ASL MRI against values acquired by ^15^O-H_2_O PET found similar reproducibilities for measurements with 25–45 days apart compared to our reproducibility for measurements with 28–49 days apart but poorer within-session reproducibility (Heijtel et al., 2014). A study using the dynamic contrast-enhanced (DCE) MRI technique found similar reproducibility for the measurement of grey and white matter separately 1 week apart compared to our whole-brain measurement (Cramer et al., 2023). Overall, the results from our study suggest that the differences in reproducibility observed in different studies are likely, at least partly, a result of the various lengths of time between examinations used in the studies.

Most studies on CBF reproducibility examine homogenous groups of healthy young adults, as was the case for this study. For example, the subjects examined had similar ages, were non-smokers and did not have any diseases or take medicine that could affect vascular function. Investigations of less homogenous groups could likely demonstrate poorer reproducibility.

### 4.2 CMRO_2_

For CMRO_2,_ we observed values similar to those acquired using invasive PET imaging (Bremmer et al., 2011; Kudomi et al., 2013) or blood sampling from jugular vein catheters (Ainslie et al., 2014). The SBO technique used to measure venous oxygen saturation in the sagittal sinus for calculation of CMRO_2_ has been validated against blood samples acquired by catheter from the jugular vein during MRI scanning (Miao et al., 2019). Reproducibility of CMRO_2_ has been far less studied than CBF, likely due to the technical difficulties in acquisition compared to CBF. A study using T2-Relaxation-Under-Spin-Tagging (TRUST) MRI for the measurement of venous oxygen saturation and PCM for CBF found reproducibility for within-session repeated measurements comparable to our study but better between-day (1–14 days) reproducibility (Liu et al., 2013). A study using calibrated BOLD imaging and a complicated calibration scheme involving inhalation of hypercapnic and hyperoxic air to quantify CMRO_2_ observed a slightly worse between day reproducibility of grey matter CMRO_2_ compared to our whole brain CMRO_2_ (Lajoie et al., 2016). The method relies on many parameters which are either fitted or derived from literature values and inaccuracies in these parameters could be the cause for the slightly poorer reproducibility.

Studies using PET imaging and inhalation of oxygen-15 as a radiotracer for CMRO_2_ measurements have demonstrated very high reproducibility for within-session repeated measurements (Coles et al., 2006). Another PET study examining CMRO_2_ using inhalation of oxygen-15 with an interval of 3 to 54 days between measurements found significantly better reproducibility than our results with 7 days or several weeks between measurements (Bremmer et al., 2011). Overall, this suggests that the PET technique for CMRO_2_ measurements has better reproducibility than using combined PCM and SBO, as in this study. However, using PET and oxygen-15 is also significantly more cumbersome, time-consuming, and invasive than MRI techniques. Generally, studies on the reproducibility of CMRO_2_ are few, and more studies using different techniques and with different timespans between examinations should be performed in the future.

### 4.3 Lactate and NAA

We observed relatively stable and reproducible concentrations for both lactate and NAA in both the occipital lobe and precuneus. Generally, we observed similar trends in correlations and reproducibility for lactate and CMRO_2_, both related to energy metabolism, suggesting that the variation in lactate and CMRO_2_ could be due to fluctuations in overall energy metabolism.

Studies on the reproducibility of MRS, similar to those on CBF and CMRO_2_, have often compared only two repeated examinations, and time variant effects were not considered. Furthermore, the various placements of the MRS voxels, the exact technique used, and the patient groups studied make it difficult to compare results.

It has been shown that NAA can be affected by brain diseases which impairs the brain metabolism, such as dementia or multiple sclerosis, however it would be expected that in healthy subjects the NAA concentration is relatively stable over time (Moffett et al., 2007). One study has demonstrated a very high intersession reproducibility of NAA with a voxel primarily located in white matter (Brooks et al., 1999). Another study using spectroscopic imaging with repeated measurements between a few days and several months apart found reasonable reproducibility of the NAA/creatine ratio in grey matter (Tedeschi et al., 1996). Studies using a 7 T MRI scanner found a reproducibility for NAA of 5.3% in the anterior cingulate cortex with 2-3 months between visits, which is better than the reproducibility we observed in the precuneus (Wijtenburg et al., 2019). The higher field strength of the MRI scanner in that study compared to ours could be a reason for the better reproducibility. Studies on lactate reproducibility are very limited; however, one study examined the reproducibility of lactate in the posterior cingulate using 7 T MRI with 1 week between measurements and found similar values to our measurement of lactate in the precuneus with 1 week between measurements (Terpstra et al., 2016).

### 4.4 Strengths and limitations

The main strength of the study is that we measured multiple parameters in the same subjects and acquired data from multiple repeated measurements at distinct time points after the initial baseline measurement. This enables us to examine time variant changes in reproducibility.

A limitation is that we only examined young healthy subjects. In cohorts of older individuals or subjects with certain diseases, the reproducibility might be lower than in young subjects. This should be considered when applying the results in projects conducting research on other subject groups. It should also be noted that all parameters in this study are measured on the same scanner with optimised MRI sequence parameters set for our purposes. Thus, this does not guarantee a direct translation to different scanners or sequence parameters, e.g., single echo pCASL instead of dual echo pCASL.

Another limitation is that we do not have a sufficient number of participants to examine which and to what extend the systemic factors explain the variation of the cerebral physiology. Systemic factors, such as SaO_2_ or arterial CO_2_ partial pressure, are known to impact CBF and changes in these parameters could account for some of the alterations in cerebral physiology over time. We observed, on average, stable values of SaO_2_, PetCO_2_, heart rate and blood pressure between the MRI sessions and a relatively narrow span of values across participants, suggesting limited effect from changes of these parameters. Nevertheless, individual natural fluctuations of systemic factors could likely still explain some of the variation in the cerebral physiology. Larger studies are needed to quantify the contributions arising from fluctuations in the various systemic factors on the cerebral physiology.

### 4.5 Conclusion

Overall, the results from the present study demonstrate satisfactorily good reproducibility of cerebral physiology measurements using non-invasive MRI techniques. We observed the best reproducibility for short timespans between the examinations and, generally, a gradual worsening of the reproducibility for longer times between measurements. Reproducibility in the context of evaluating an intervention or disease evolution should therefore be estimated based on the time scale of the study.

## Data Availability

The datasets presented in this article are not readily available because of privacy restrictions. The parameters derived from the MRI-images and supporting data are available upon reasonable request. Requests to access the datasets should be directed to mark.bitsch.vestergaard@regionh.dk.

## Abbreviations

A-V.O_2_, Arteriovenous oxygen saturation difference; ASL, Arterial spin labelling; CBF, Cerebral blood flow; CMRO_2_, Cerebral metabolic rate of oxygen; CoV_ws_, Within-subject coefficient of variation; CSF, Cerebrospinal fluid; FOV, Field of view; LoA, Limit of agreement; MRS, Magnetic resonance spectroscopy; NAA, N-acetyl aspartate; pCASL, Pseudo-continuous arterial spin labelling; PCM, Phase contrast mapping; PET, positron emission tomography; SBO, susceptibility-based oximetry; SaO_2_, oxygen saturation of the arterial blood; SvO_2_, oxygen saturation of the venous blood; TD, Saturation delay; TE, Echo time; TI, Inversion time; TR, Repetition time.
