# Supplementary material for: Reproducibility of cerebral blood flow, oxygen metabolism, and lactate and N-acetyl-aspartate concentrations measured using magnetic resonance imaging and spectroscopy
Source: Front Physiol. 2023 Sep 5;14:1213352. doi: 10.3389/fphys.2023.1213352 (PMC10508186; doi:10.3389/fphys.2023.1213352)
Supplement: Supplementary file 1 [file DataSheet1.pdf]

Supplementary material

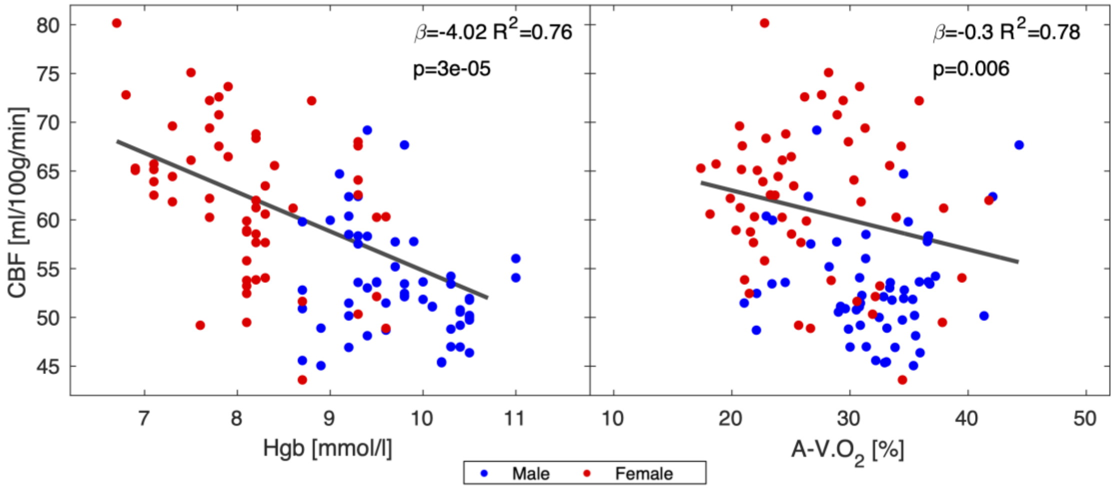

Supplementary figure 1. Correlation between cerebral blood flow (CBF) and haemoglobin (Hgb) and arteriovenous oxygen saturation difference (A-V.O_2) across all measurements. CBF correlated significantly with both Hgb concentration and (A-V.O_2).

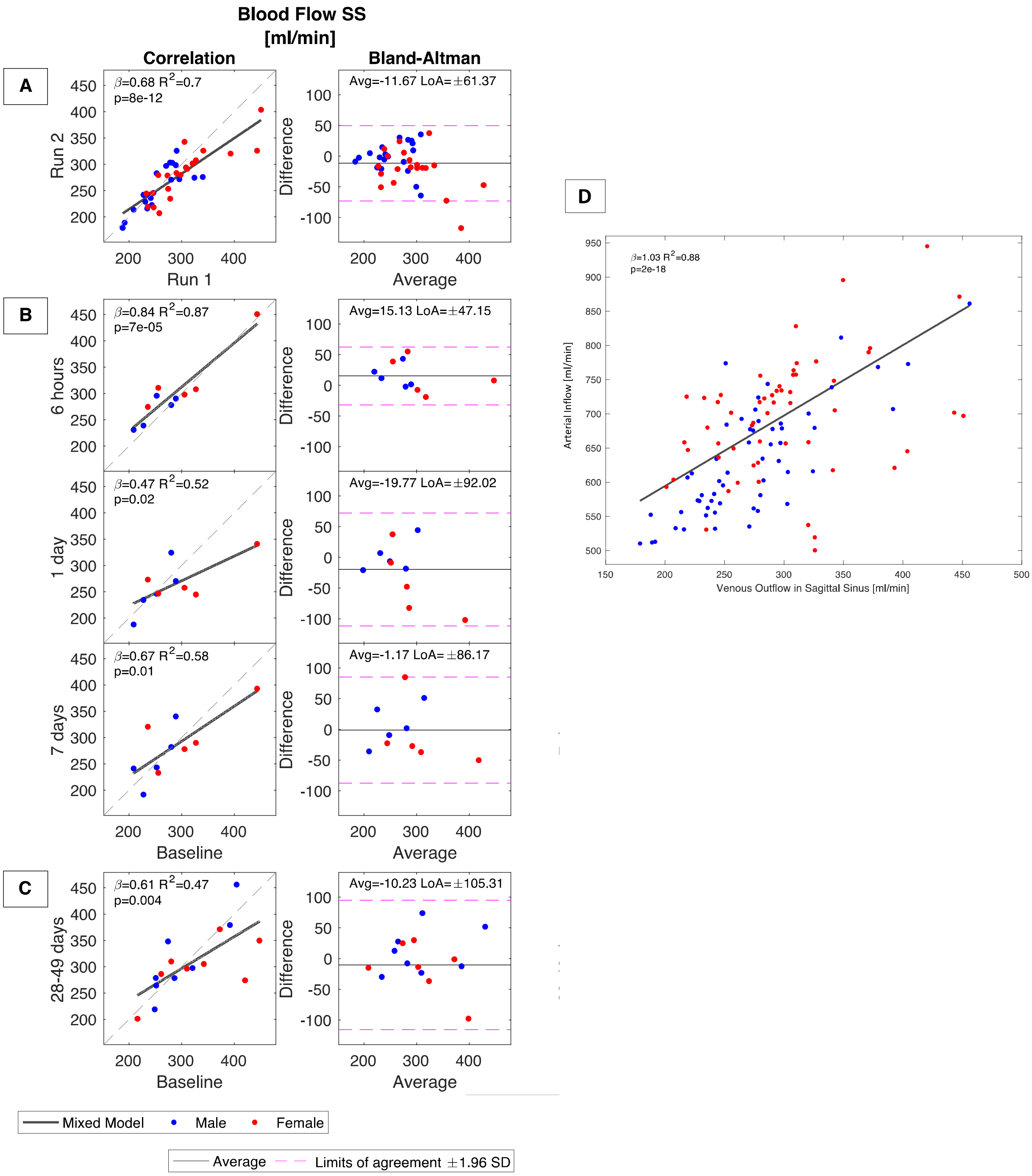

Supplementary figure 2. Correlation and reproducibility between baseline measurements and the subsequent acquisitions of blood flow in the sagittal sinus (SS). The within-session correlation is shown in (A). The correlations between baseline values and values from each subsequent MRI session completed 6 hours, 1 day and 7 days after the initial MRI session from Group A are shown in (B). The correlations between baseline and subsequent MRI sessions from

Group B are shown in (C). The regression slopes (β), R^2 coefficients, and p values from the regressions and limits of agreement (LoAs) are noted in each panel. A general pattern of weaker correlation and wider limit of agreement with increasing time between sessions is noted.

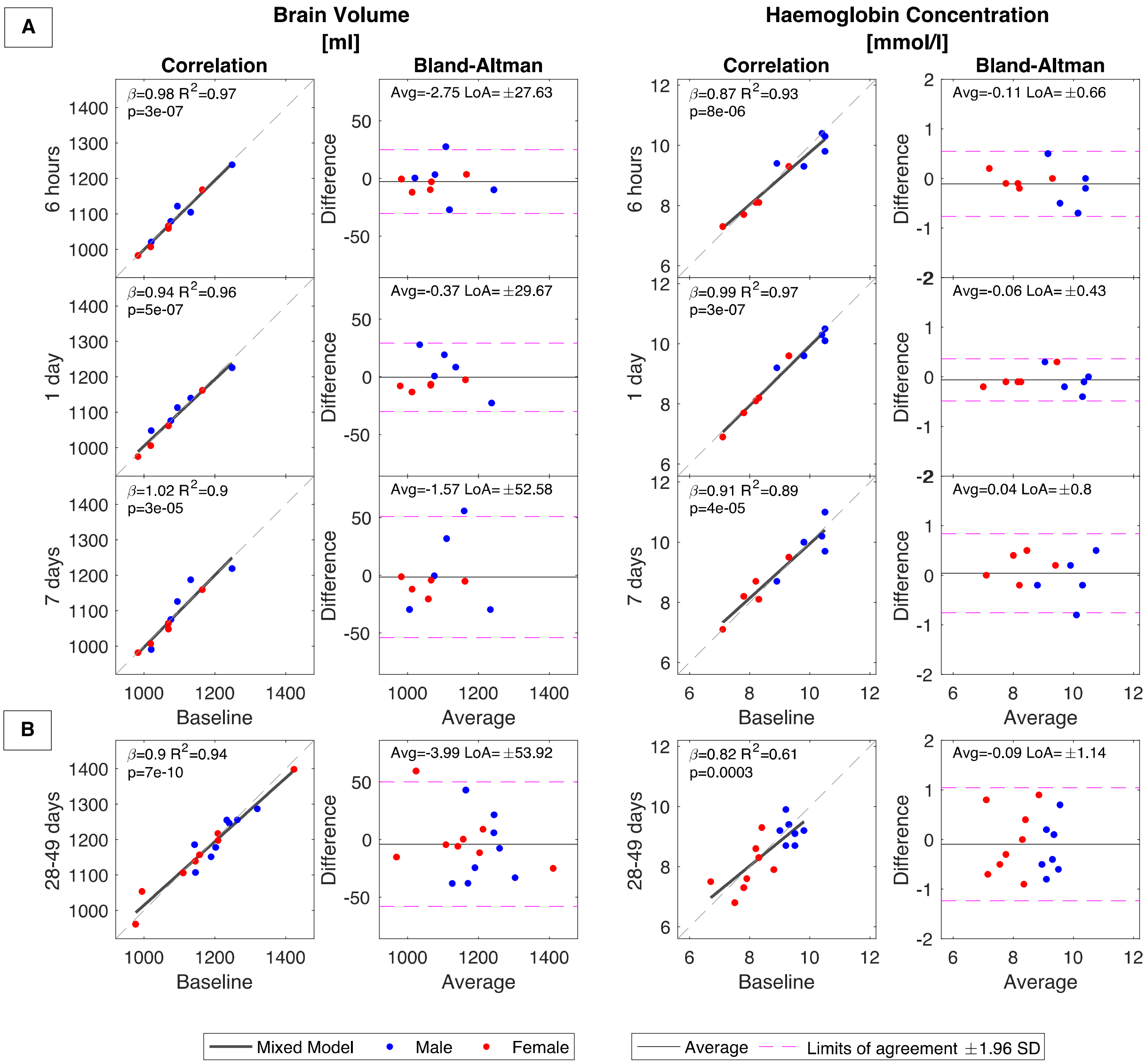

Supplementary figure 3. Correlation and reproducibility between baseline measurements and the subsequent acquisitions of total brain volume and haemoglobin concentrations. The correlations between baseline values and values from each subsequent MRI session completed 6 hours, 1 day and 7 days after the initial MRI session from Group A are shown in (A). The correlations between baseline and subsequent MRI sessions from Group B are shown in (B).

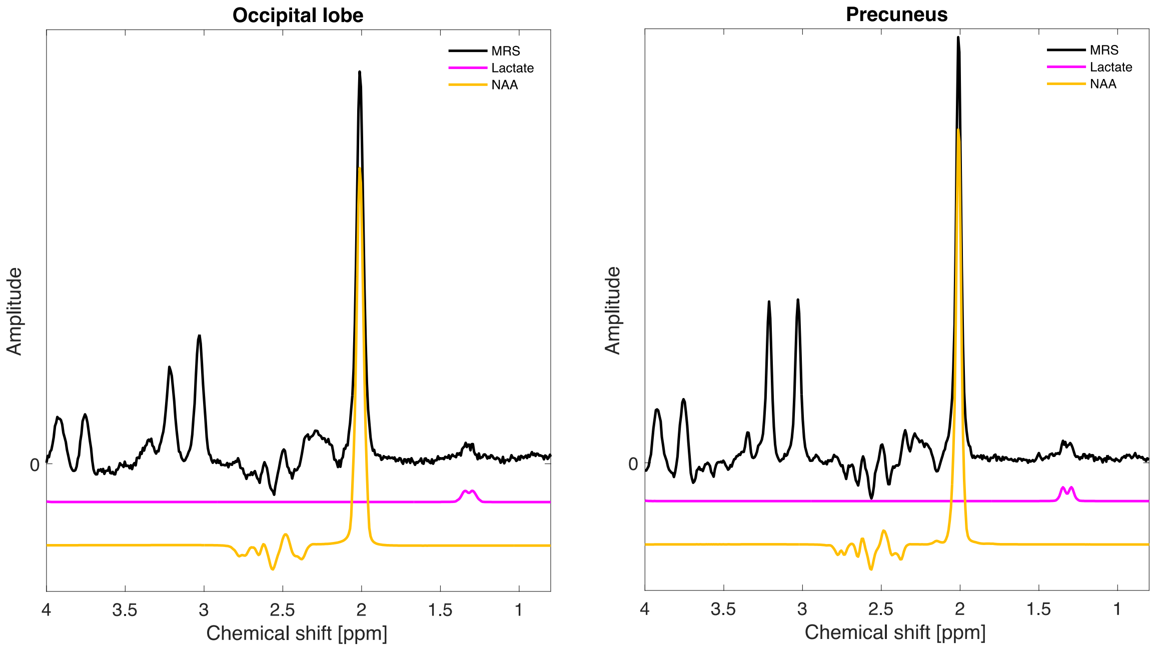

Supplementary figure 4. Example of MRS spectra measured in the occipital lobe and precuneus with corresponding fits of the lactate and N-acetyl-aspartate (NAA) peaks. The spectra were measured using a single-voxel water-suppressed point-resolved 1H-spectroscopy (PRESS) sequence (TE/TR = 288/2000 ms; voxel size = 30 x 35 x 30 mm^3; 176 averages, 1024 data points).
